# Blue laser light inhibits biofilm formation in vitro and in vivo by inducing oxidative stress

**DOI:** 10.1038/s41522-019-0102-9

**Published:** 2019-10-09

**Authors:** Katia Rupel, Luisa Zupin, Giulia Ottaviani, Iris Bertani, Valentina Martinelli, Davide Porrelli, Simone Vodret, Roman Vuerich, Daniel Passos da Silva, Rossana Bussani, Sergio Crovella, Matthew Parsek, Vittorio Venturi, Roberto Di Lenarda, Matteo Biasotto, Serena Zacchigna

**Affiliations:** 1 Department of Medical, Surgical and Health Sciences, University of Trieste, 34127 Trieste, Italy; 2 Bacteriology Laboratory, International Centre for Genetic Engineering and Biotechnology (ICGEB), 34149 Trieste, Italy; 3 Cardiovascular Biology Laboratory, International Centre for Genetic Engineering and Biotechnology (ICGEB), 34149 Trieste, Italy; 4 Department of Microbiology, University of Washington, Seattle, WA 98195 USA; 5 Institute for Maternal and Child Health-IRCCS “Burlo Garofolo”, 34137 Trieste, Italy

**Keywords:** Biofilms, Dentistry

## Abstract

Resolution of bacterial infections is often hampered by both resistance to conventional antibiotic therapy and hiding of bacterial cells inside biofilms, warranting the development of innovative therapeutic strategies. Here, we report the efficacy of blue laser light in eradicating *Pseudomonas aeruginosa* cells, grown in planktonic state, agar plates and mature biofilms, both in vitro and in vivo, with minimal toxicity to mammalian cells and tissues. Results obtained using knock-out mutants point to oxidative stress as a relevant mechanism by which blue laser light exerts its anti-microbial effect. Finally, the therapeutic potential is confirmed in a mouse model of skin wound infection. Collectively, these data set blue laser phototherapy as an innovative approach to inhibit bacterial growth and biofilm formation, and thus as a realistic treatment option for superinfected wounds.

## Introduction

The availability of antibiotics has revolutionized modern medicine and dramatically improved the survival of patients affected by bacterial infections, as well as the success of a wide range of interventions, including surgery, organ transplantation, and chemotherapy. Unfortunately, both the overuse and misuse of these compounds has resulted in the massive spread of antibiotic resistance among common bacterial pathogens. In addition, over the last decades, the pharmaceutical industry has not invested significant resources in the discovery and development of novel antimicrobial drugs, further limiting the efficacy of currently available antibiotic therapies. Consistently, the World Health Organization has included antibiotic resistance among the three most important public health threats of the 21st century.^1^ It is estimated that in Europe 25,000 people die each year as a result of multidrug-resistant bacterial infections, resulting in a yearly cost of €1.5 billion for the economy of the European Union.^2^

Antibiotic resistance is increasing among bacteria that cause common infections, including pneumonia, urinary tract, cutaneous, and mucosal infections. Several nosocomial infections are caused by highly resistant bacteria, involving methicillin-resistant *Staphylococcus aureus* (MRSA) and multidrug-resistant gram-negative bacteria, such as *Pseudomonas aeruginosa*. These infections are particularly aggressive in immunocompromised and oncological patients, negatively impacting on their prognosis.^3,4^

An emerging, novel approach to control antibiotic-resistant bacterial infections is based on the use of light, in particular of blue wavelengths (400–470 nm).^5,6^ While most of the spectra at high irradiance are able to kill bacteria by a photothermal effect,^7^ exposure to blue light has shown efficacy in decreasing the viability of various bacterial species, including *Pseudomonas aeruginosa*, *Porphyromonas gingivalis*, *Helicobacter pylori*, and methicillin-resistant *Staphylococcus aureus* (MRSA)^8,9^ even at the low irradiance.

The mechanism by which the blue light can exert an antimicrobial activity is largely unknown and probably relies on a photochemical, rather than a photothermal effect. It has been proposed, but never demonstrated, that the interaction of blue light with endogenous photosensitizing molecules, possibly porphyrins and flavins, determines the generation of reactive oxygen species (ROS). Absorption of blue light is expected to bring porphyrin molecules into an excited triplet state, leading to a non-radiative transfer of energy to the chemically stable molecular oxygen. The excited oxygen molecule, dissociated into two highly reactive atomic species, may damage the integrity of cells, eventually leading to microbial death.^10^

The capacity of blue light to contrast bacterial infections at low power and energy densities opens novel opportunities to safely apply this strategy to patients, avoiding the major side effects caused by the photothermal effect, namely burn lesions on treated areas. A recent study showed that a 415 nm LED light applied on mouse burns infected with *P. aeruginosa* was not toxic for the skin and resulted in an improved clearance of bacterial cells and overall survival of the animals.^11^ While the most common sources of blue light are LEDs, emerging evidence indicates that blue laser devices exert a similar antimicrobial efficacy, at least against MRSA.^12,13^

Based on these considerations, we examined the potential of blue laser light to eradicate infections by *P. aeruginosa*, which is considered a model of antibiotic-resistant bacteria.^14^ Here, we show that this strategy is feasible, effective, and safe, both in vitro an in vivo.

## Results

### Blue laser light exerts direct antimicrobial activity on *P. aeruginosa* grown in planktonic state, on solid surfaces and as biofilms

**Fig. 1:**
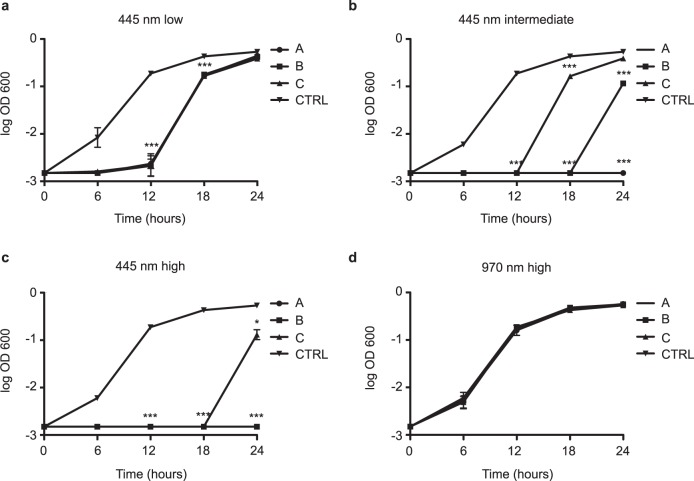
Effect of blue and infrared laser light on *P. aeruginosa* planktonic growth. **a** All protocols using blue light and characterized by a fluence of 40 J/cm^2^ but different irradiances (A,B,C-low) inhibited bacterial growth up to 12 h after irradiation (*p* < 0.0001 compared to CTRL). **b** All protocols using blue light and characterized by a fluence of 60 J/cm^2^ but different irradiances (A,B,C-intermediate) inhibited bacterial growth up to 24 h after irradiation, as no difference in bacterial density over time could be detected (Friedman test for differences in bacterial growth over time *p* < 0.0001 only in CTRL). While protocol A-intermediate completely suppressed bacterial growth up to 24 h (*p* < 0.0001 compared to CTRL), the other protocols allowed bacterial growth after 18 h (Protocol C-intermediate) or 24 h (Protocol B-intermediate). **c** All protocols using blue light and characterized by a fluence of 120 J/cm^2^ but different irradiances (A,B,C-high) inhibited bacterial growth up to 24 h after irradiation, as no difference in bacterial density over time could be detected (Friedman test for differences in bacterial growth over time *p* < 0.0001 only in CTRL), with a minimal increase in bacterial density observed at 24 h for protocol C, significantly lower compared to untreated samples (Fig. 1c, *p* < 0.01). **d** None of the protocols using infrared laser and characterized by a fluence of 120 J/cm^2^ but different irradiances (A,B,C-high) showed any antimicrobial activity compared to untreated samples (CTRL). Data are shown as mean ± SD. *ANOVA two-way *p* < 0.01; ***ANOVA two-way *p* < 0.0001

Planktonic cultures of *P. aeruginosa* were exposed to laser light at different wavelength, fluence, peak power, and irradiance (Table 1), and their growth was monitored over 24 h. As shown in Fig. 1a–c, all protocols delivering blue laser light effectively reduced bacterial growth, although to different extents. Protocols A,B,C-low, characterized by a fluence of 40 J/cm^2^, significantly inhibited bacterial growth up to 18 h, independent of the irradiance (Fig. 1a, *p* < 0.0001 for all protocols at T12 and T18). After 24 h, bacterial density was comparable to untreated samples. At 60 J/cm^2^ all tested protocols significantly inhibited bacterial growth up to 12 h (*p* < 0.0001 for protocols A,B,C-intermediate). This inhibition persisted 24 h in the case of protocol A (*p* < 0.0001 compared to CTRL), whereas in the other cases we observed an increase in bacterial density at T18 and T24, when bacterial density in protocol C was comparable to the untreated samples (Fig. 1b). Protocols A,B,C-high, reaching a fluence of 120 J/cm^2^, almost completely inhibited bacterial growth, with an only a minimal increase in bacterial density observed at 24 h for protocol C, but significantly lower compared to untreated samples (Fig. 1c, *p* < 0.0001 for protocols A,B,C-high compared to CTRL at all time points, except for C-high at T24, *p* < 0.01). Differences in bacterial growth among treated and untreated samples at 6 h were minimal due to a prolonged lag phase in all samples, due to slow growth conditions in microtiter plates. The prolonged lag phase and the steeper growth curves seen in protocols A,B,C-low, B,C-intermediate, and C-high are probably caused by a depletion in the number of viable bacteria in the irradiated wells, although a laser-mediated bacteriostatic effect cannot be excluded.

**Table 1:** Classification of irradiation protocols

Protocol name	Irradiance at sample (W/cm^2^)	Fluence (J/cm^2^)
A	≤0.30	40 = low
		60 = intermediate
		120 = high
B	0.31–0.60	40 = low
		60 = intermediate
		120 = high
C	≥0.61	40 = low
		60 = intermediate
		120 = high

We then wanted to assess whether these effects were specifically exerted by the blue laser light or could be reproduced by other wavelengths commonly used in medicine. Therefore, we repeated the experiments using the most effective protocols (A,B,C-high) applying a 970 nm laser light. None of these treatments induced a significant change in bacterial growth, indicating absence of antimicrobial activity exerted by this wavelength (Fig. 1d). Since blue laser protocols C-low, intermediate, and high were poorly effective in antimicrobial activity, were excluded from further experimentation.

**Fig. 2:**
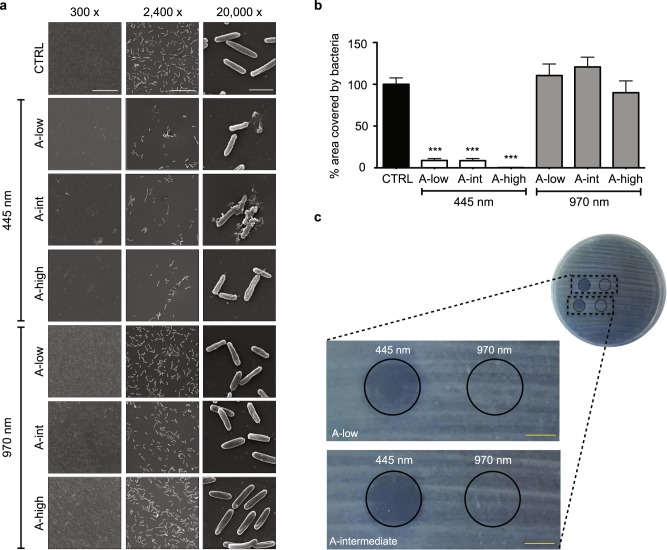
Effect of blue and infrared laser light on *P. aeruginosa* cells on solid substrates. **a** Representative SEM images at different magnifications of bacteria treated with protocols A-low, A-intermediate and A-high, using either blue or infrared laser light (scale bars: left = 200 μm, middle = 20 μm, right = 3 μm). **b** Quantification of the area covered by bacteria in each SEM image. Data are shown as mean ± SD. ***Mann–Whitney *U* test treatment versus control *p* < 0.0001. **c** Representative images of an agar plate showing inhibition of *P. aeruginosa* growth (gray spot) upon irradiation with blue but not infrared laser protocols A-low and A-intermediate (scale bars = 10 mm)

We also performed Scanning Electron Microscopy (SEM) on planktonic bacteria seeded on a sterile surface and exposed to protocols A-low, intermediate, and high at either 445 nm or 970 nm. We confirmed that blue laser irradiation protocols significantly reduced bacterial density (*p* < 0.0001 for all protocols), whereas the infrared light did not cause any difference compared to untreated samples (Fig. 2a, b). High magnification images of blue-irradiated samples showed severely damaged bacterial cells, with disrupted outer membranes interrupted by multiple blebs. These changes were never observed upon treatment of bacteria with 970 nm light (Fig. 2a). Thus, blue laser light importantly compromises bacterial integrity and viability.

On agar plates, protocols A-low and A-intermediate delivering blue laser light completely inhibited bacterial growth inside the spot size of irradiation, which was surrounded by a homogeneous layer of growing bacteria (Fig. 2c). In agreement with the results obtained in planktonic cultures, the 970 nm laser light did not exert any inhibition on bacterial growth (Fig. 2c).

Since one of the key factors responsible for the resistance of *P. aeruginosa* to both antibiotics and immune cells is its capacity to grow as biofilms, the development of innovative approaches able to disrupt biofilm structure is of great importance. We therefore assessed the potential of blue laser light to simultaneously disrupt existing biofilms and prevent their generation, using two models for in vitro biofilm analysis,^15^ in microtiter plates and flow cells.

**Fig. 3:**
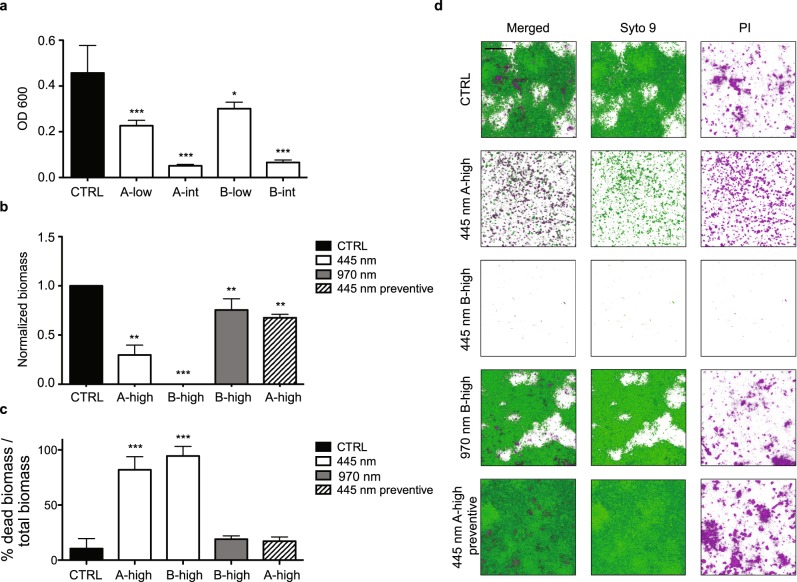
Effect of blue and infrared laser on *P. aeruginosa* biofilms. **a** Quantification of *P. aeruginosa* growth as biofilms on microtiter plates. ***Mann–Whitney *U* test treated vs. CTRL *p* < 0.0001. *Mann–Whitney *U* test treated vs. CTRL *p* < 0.01. **b** Biofilm biomass normalized on control samples upon treatment with blue laser protocol A-high, both in young (445 nm preventive) and established biofilm, and protocol B-high, delivered either with blue or infrared laser light. **Mann–Whitney *U* test *p* < 0.001. ***Mann–Whitney *U* test *p* < 0.0001. **c** Portion of dead biomass on total biofilm biomass upon treatment with blue laser protocol A-high, both in young (445 nm preventive) and established biofilm, and protocol B-high, delivered either with blue or infrared laser light. ***Mann–Whitney *U* test *p* < 0.0001. **d** Representative images of untreated biofilm and different treatment conditions, stained with Syto9 and PI and visualized using Laser Scanning Confocal Microscopy (scale bar = 40 μm). Data are shown as mean ± SD

We tested four different blue laser protocols (A-low, A-intemediate, B-low, and B-intermediate) on *P. aeruginosa* biofilms grown in Calgary devices and found that all tested protocols significantly reduced biofilm growth (*p* < 0.0001 for A-low, A-intermediate, and B-intermediate, *p* < 0.01 for B-low), with protocols A-intermediate and B-intermediate being able to completely eradicate bacterial biofilm (Fig. 3a).

We confirmed these results using flow cells in conjunction with Laser Scanning Confocal Microscopy, which allows performing live imaging and monitoring the formation and development of bacterial biofilms. Figure 3d shows representative images of biofilms, in which the whole biomass in stained in green (Syto9) and dead cells are stained with PI (Propidium Iodide) in purple.

In untreated biofilms, most of the bacteria are alive, with a few, randomly distributed spots of cell death corresponding to regions of poor oxygen and nutrients content, as well as eDNA released at the base of the aggregates^16,17^ (Fig. 3d).

Irradiation of the mature biofilm with blue laser protocol A-high (with an irradiance ranging from 0.3 W/cm^2^ to 0.15 W/cm^2^ from the top to the bottom of the biofilm) markedly reduced biofilm biomass, due to detachment of the cells from the surface, and the ones that remained on the surface were mostly stained with PI (Fig. 3b–d). We then further increased the irradiance up to 0.6 W/cm^2^ and 0.3 W/cm^2^ at the top and bottom of the biofilm (protocol B-high). In these conditions, the whole biomass almost completely detached from the surface and was washed away with no living bacteria detected (Fig. 3b–d). When this latter protocol was delivered using an infrared laser source, a reduction in biomass was detected (Fig. 3b–d), although not as drastic as observed in samples exposed to blue laser and the remaining cells presented similar levels of PI staining of those without treatment. Three-dimensional representation of biofilms is provided in Supplementary Movies S1–S4.

Finally, we assessed the capacity of blue laser light to prevent biofilm formation. For this purpose, the flow cell was irradiated shortly using protocol A-high after the attachment period and biofilm formation was monitored over time. A reduction in bacterial density compared to the untreated cells was clearly visible at 24 h and persisted over the following three days. At 72 h, bacteria that survived were able to generate a smaller and less mature biofilm than the one formed by untreated bacterial cells. We then finally stained the biofilms to quantify both the biomass and eDNA, corresponding to dead cells, as described above. While the percentage of dead biomass did not differ between the two groups, the biomass of the treated biofilm was significantly reduced by preventive blue laser irradiation (Fig. 3b–d and Supplementary Movie S5).

### The antimicrobial activity of blue laser light on *P. aeruginosa* relies on the generation of oxidative stress

In order to begin to understand the mechanism by which blue laser exerts its antimicrobial activity, we evaluated the possible occurrence of a photothermal effect at the site of irradiation. We applied increasing values of irradiance (Protocols A,B,C, Supplementary Fig. 1a–c) on Petri dishes containing LB agar and measured a progressive but modest increase in the temperature at the site of irradiation, albeit always compatible with *P. aeruginosa* viability (which significantly decreases at temperatures higher than 60 °C^18^).

To confirm that the antimicrobial activity depends on the blue wavelength, we exposed planktonic bacteria to protocols A-intermediate and A-high, in which the blue light was emitted by a LED source, and determined their growth, as described in the Methods section. Protocol A-intermediate significantly inhibited bacterial growth (*p* < 0.001 at 12, *p* < 0.0001 at 18 and 24 h). At a fluence of 120 J/cm^2^, protocol A-high markedly inhibited bacterial growth over time, with only a minimal residual increase in bacterial density observed at 24 h (*p* < 0.0001 at 12, 18, and 24 h; Supplementary Fig. 2). When comparing the efficacy of blue light delivered by either a laser or a LED source, we found that laser light was significantly more effective when using protocol A-intermediate after 24 h (*p* < 0.05), but not when using protocol A-high, which completely killed all bacterial cells independent of the light source (Supplementary Fig. 2).

**Fig. 4:**
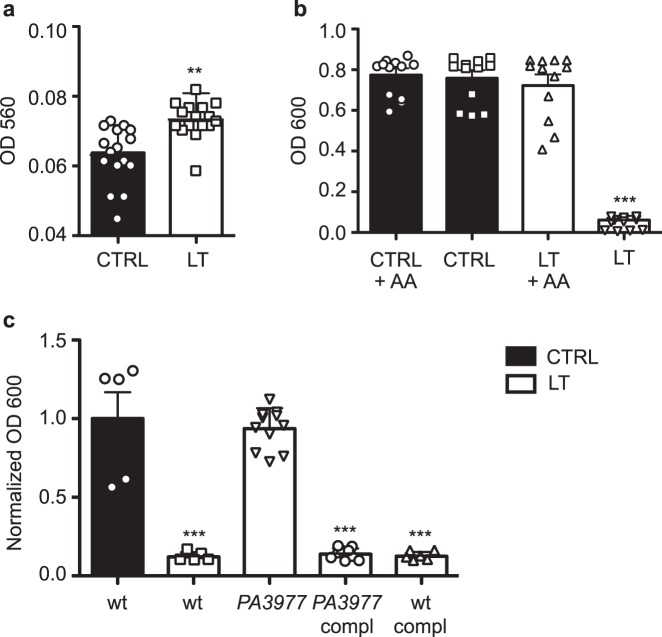
Oxidative stress mediates the antimicrobial action of blue light. **a** Quantification of TOS levels in control (CTRL) and blue laser-treated (LT) bacteria (protocol A-intermediate). **Mann–Whitney *U* test *p* < 0.001. **b** Bacterial growth after laser treatment (LT, protocol A-intermediate), either in the presence or in the absence of ascorbic acid (AA). ***Mann–Whitney *U* test *p* < 0.0001. **c** Effect of blue laser treatment (LT, protocol A-intermediate) on the growth of wild-type (wt) *P. aeruginosa*, *PA3977* knock-out mutants, *PA3977* mutants complemented with a *PA3977* plasmid, wt bacteria transformed with the same *PA3977* plasmid. Data are reported normalized on untreated wt. ***Mann–Whitney *U* test *p* < 0.0001. Data are shown as mean ± SD

We then assessed whether blue laser light induces oxidative stress, which has been proposed among the possible mechanisms by which this wavelength can inhibit the growth of bacterial species.^5,6,11^ We measured the Total Oxidant Status (TOS) after irradiation of bacterial suspensions and detected a significant increase already at 1 h after exposure to blue laser light, as shown for protocol A-intermediate in Fig. 4a (Mann–Whitney *U*-test *p* < 0.001). Subsequently, we confirmed the involvement of ROS production by evaluating the antimicrobial activity of blue laser light in the presence of ascorbic acid (40 μM), a natural ROS scavenger. As shown in Fig. 4b, the presence of ascorbic acid totally rescued *P. aeruginosa* viability and growth, without inducing any change in the growth of not irradiated cells.

The molecular targets for blue light are currently unknown, possible intracellular photoreceptor candidates are the porphyrins, which contain functional groups that absorb light generating ROS.^6^ Therefore, we irradiated a *P. aeruginosa* genomic knock-out mutant strain (*PA3977*) using protocol A-intermediate, lacking the aminotransferase involved in the initial phases of porphyrin biosynthetic pathway (hemL). This mutant produces less porphyrins compared to wild-type (wt) cells and appeared to be more resistant to blue laser irradiation, growing at a rate comparable to the one of wt untreated cells. This was a first indication that porphyrins are likely targets for blue light in bacterial cells. To definitely prove this hypothesis, we complemented *PA3977* mutant bacteria with a plasmid carrying the complete sequence of the *PA3977* open reading frame together with its ribosome binding site. Complemented bacteria returned sensitive to blue laser irradiation, thus confirming a crucial role of hemL and the porphyrin biosynthetic pathway in the response of microbial cells to blue laser light (Fig. 4c).

### Bactericidal protocols delivering blue laser light exert minimal toxicity on human keratinocytes

**Fig. 5:**
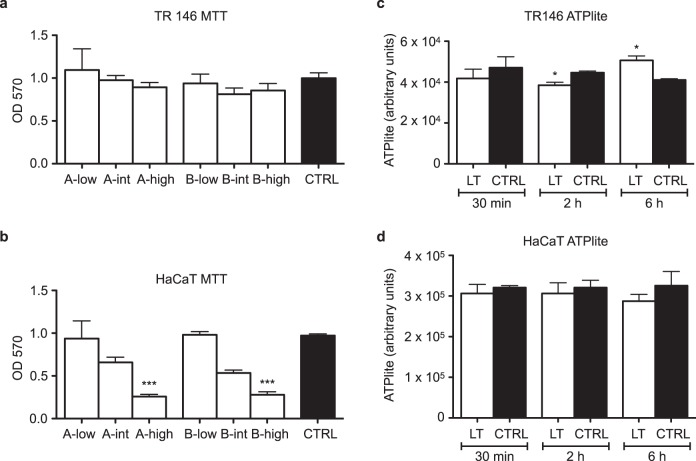
Blue laser light can be safely applied to human epithelial cells. **a** Quantification of the viability of TR146 oral keratinocytes upon exposure to the indicated blue laser protocols using the MTT assay. **b** Quantification of the viability of HaCaT skin keratinocytes upon exposure to the indicated blue laser protocols using the MTT assay (***Mann–Whitney *U* test *p* < 0.0001 compared to CTRL). **c** Quantification of the metabolic activity of TR146 oral keratinocytes upon blue laser treatment (LT, protocol A-intermediate) using the ATPlite assay (*Mann–Whitney *U* test *p* < 0.05 compared to CTRL). **d** Quantification of the metabolic activity of HaCaT skin keratinocytes upon blue laser treatment (LT, protocol A-intermediate) using the ATPlite assay. Data are shown as mean ± SD

To start exploring whether this antimicrobial activity of blue laser light can be exploited to treat bacterial infections in vivo, we tested the most effective protocols for their capacity to induce any possible toxic effect in eukaryotic cells. As the blue light has a poor penetration capacity through biological tissues and its effects are limited to the most superficial epidermal layers,^19^ we irradiated cells derived from both the human oral mucosa and skin, assuming that the most plausible application of this approach could be the treatment of mucosal and cutaneous infections. We first tested the various blue laser protocols on TR146 mucosal cells, derived from a well-differentiated keratinizing squamous cell carcinoma and found that their viability was not affected by any of the tested protocols (Fig. 5a, Kruskal-Wallis *p* = NS). The same protocols were also applied to HaCaT cells, a spontaneously transformed aneuploid immortal keratinocyte cell line. These cells were more susceptible to blue laser irradiation, with a progressive reduction in cell viability along with the increase in fluence and irradiance. While cell viability was not significantly affected by low and intermediate fluences (40 and 60 J/cm^2^ respectively), at the highest fluence (120 J/cm^2^), HaCaT cells died using either of the two power densities (protocols A-high and B-high, *p* < 0.0001, Fig. 5b).

We also assessed the effect of the same protocols on cell metabolism, by measuring the levels of intracellular ATP in either epithelial type upon blue laser exposure. Only modest changes in ATP levels were observed in TR146 cells, which showed a decrease at 2 h and an increase at 6 h, with no apparent correlation with cell viability. No change was observed in HaCaT cells at any of the considered time points (Fig. 5c, d).

### Blue laser irradiation significantly reduces bacterial load in a mouse model of *P. aeruginosa* cutaneous infection

**Fig. 6:**
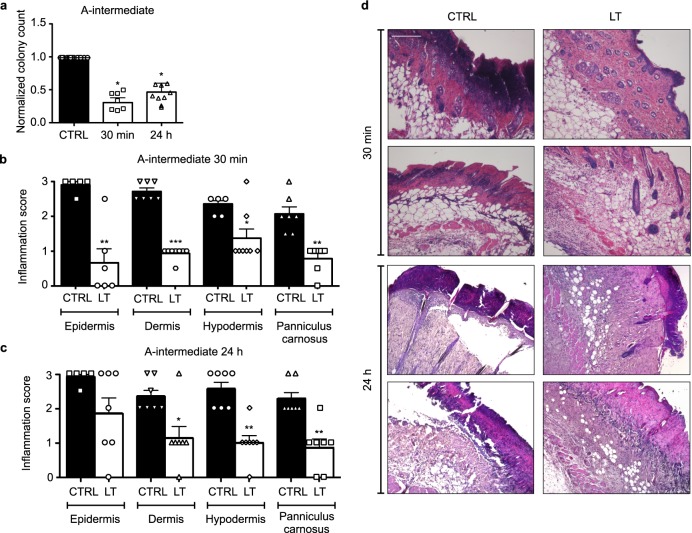
Blue laser light exerts antimicrobial activity on infected wounds in vivo. **a** Normalized quantification of bacterial load in control (CTRL) and blue laser-treated (LT) samples either 30 min or 24 h after the infection. *Mann–Whitney *U* test *p* < 0.05. **b** Quantification of the inflammation score in the indicated skin layers in control (CTRL) and blue laser-treated (LT) samples 30 min after infection. ***Mann–Whitney *U* test *p* < 0.0001, **Mann–Whitney *U* test *p* < 0.001 and *p < 0.05. Data are shown as mean ± SD. **c** Quantification of the inflammation score in the indicated skin layers in control (CTRL) and blue laser-treated (LT) samples 24 h after infection. **Mann–Whitney *U* test *p* < 0.001 and *p < 0.05. Data are shown as mean ± SD. **d** Representative images of histological samples of the skin of control (CTRL) and blue laser-treated (LT) mice, irradiated either 30 min or 24 h after infection (scale bar = 200 μm). Data are shown as mean ± SD

It was also of interest to test the antimicrobial efficacy of blue laser light irradiation in vivo in a mouse model of *P. aeruginosa* cutaneous wound infection in young and established biofilms. As shown in Supplementary Fig. 3, mice were shaved on their right flank and a bacterial suspension containing 10^6^ CFU was topically applied on a squared skin abrasion. After 30 min in the first group and 24 h in the second group, protocol A-intermediate was applied to half of the animals, which were sacrificed after additional 24 h for quantification of bacterial load and histological analysis. Laser-treated wounds were colonized by a significantly lower amount of bacterial cells compared to controls (Fig. 6a, *p* < 0.01 in both groups). Histological evaluation of the skin samples showed a diffuse reduction of the epithelial thickness in all samples, consistent with the abrasions. In infected control samples, the skin was markedly infiltrated by inflammatory cells in all its layers. In addition, when mice were sacrificed 48 h after infection, a detachment of epidermal layer from the dermal one was detected in all samples (Fig. 6d). In contrast, laser-treated samples showed modest or absent inflammation, with preservation of the normal skin architecture, even in mice treated the day after infection (Fig. 6d). Quantification of the severity of inflammation, following a score described in Table 2, showed a reduction in the area infiltrated by inflammatory cells in all the layers composing the mouse skin in both groups (*p* < 0.05 in all cases with the exception of the epidermal layer in the group treated 24 h after infection; Fig. 6b, c).

**Table 2:** Semi-quantitative score for quantification of inflammation in histological samples

Severity score	Description
0	Absence of inflammation
1	Modest inflammation (<5% area covered by inflammatory cells)
2	Moderate inflammation (5–50% area covered by inflammatory cells)
3	Severe inflammation (>50% area covered by inflammatory cells)

## Discussion

This work provides significant evidence of the capacity of the 445 nm (visible blue) laser light to exert direct antimicrobial activity, without the addition of any exogenous photosensitizing agent, against the important human opportunistic pathogen *P. aeruginosa*.^20^ While a few previous studies have in part investigated some aspects of this effect, this compares for the first time the efficacy of multiple protocols on bacterial biofilms in culture and in vivo, also shedding light on the molecular mechanisms responsible for the observed antimicrobial activity. This appears of particular relevance for a rational and evidence-based translation of the use of blue light to combat bacterial biofilm in the clinics.

While all our data support the antimicrobial effect of blue laser light, we noticed a different efficacy of the tested protocols depending on the growth conditions of bacterial culture. For instance, while most of the protocols delivering a fluence of 40 J/cm^2^ only partially and transiently inhibited the growth of planktonic bacteria (Fig. 1a), they efficiently worked on agar plates (Fig. 2c). This could be due to the low penetration potential of blue light, which is very effective on a superficial monolayer of bacterial cells, as those seeded on agar, but is partially absorbed and diffracted in a bacterial suspension. The high efficacy on the surface of solid substrates prompts additional studies exploring the usefulness of blue laser as an alternative to UV light to sterilize exposed surfaces in non-medical, industrial settings. Conversely, higher fluences (at least 60 J/cm^2^) are required to exert a significant antimicrobial activity on planktonic bacteria. This hypothesis is also supported by the evidence that the efficacy of blue LED light to inhibit the growth of MRSA depends on bacterial density.^21^ While literature data is not always easy to compare due to the use of different protocols and experimental settings, our results are consistent with previous studies, adopting a similar culture set-up on 96-well plates, which achieved *P. aeruginosa* eradication at a fluence of 50 J/cm^2^.^22^

One of the key elements underlying *P. aeruginosa* pathogenicity and resistance to both antibiotics and immune cells is its capacity to growth as biofilms; these are complex structures in which bacterial communities are embedded in a dense extracellular matrix. Within biofilms, bacteria secrete virulence factors able to neutralize and kill polymorphonuclear leukocytes, thus allowing the persistence of chronic infections even in the presence of apparently adequate antibiotic therapy.^23–25^ Therefore, any innovative antimicrobial strategy should also be able to disrupt mature biofilms and/or prevent their formation. Biofilms are difficult to standardize in culture, thus the reliable assessment of the effectiveness of any antimicrobial treatment on biofilm formation is challenging. Among the various in vitro biofilm growth methods,^26^ microtitre plate assays and flow cells are the most commonly used.^15^ We adopted both assays to evaluate the effect of blue laser irradiation, applying the most effective protocols on planktonic bacteria. Also in this case, efficient biofilm disruption could be achieved only with protocols delivering higher fluences (protocols A-intermediate and B-intermediate), consistent with the data described in the only previous study investigating the effect of blue light on biofilm grown on Calgary devices.^27^ Higher irradiances and fluences were required to eradicate microbial cells and reduce/disrupt the biomass of biofilms grown in flow cells (protocols A-high and B-high). This method showed that a single irradiation with blue laser significantly reduced biomass formation up to 72 h after treatment, in accordance with recent results obtained using LED sources.^28,29^ Although it is currently unknown whether this effect is due to either reduced cell viability and subsequent delay in biofilm development or a direct effect of blue light on specific intra/extracellular signaling pathways essential for biofilm formation, this is the first evidence of the antimicrobial and anti-biofilm effect of blue light using the flow cell technique.

Our experiments indicate that the antimicrobial effect is specific for the blue wavelengths, as we could not reproduce its effects by using an infrared laser light (970 nm) in any of the tested assays. In contrast, previous studies have reported significant inhibition of *P. aeruginosa* growth using different wavelengths in the 660–904 nm range.^30,31^ This could be due either to the use of higher irradiances, inducing significant photothermal toxicity, or that the endpoint was not eradication but rather a reduction of bacterial growth, which would be of limited therapeutic potential.

What are the molecular mechanisms responsible for the antimicrobial and anti-biofilm effect of blue laser light? While excluding a photothermal effect, our data suggest the blue wavelength might target specific molecular substrates. As also shown by others,^8,9^ the same irradiation protocols delivered using a LED source similarly reduced bacterial growth, although with less efficacy compared to laser light (Supplementary Fig. 2a, b), possibly due to the wider range of wavelengths, lack of coherence, directionality, and collimation. Especially longitudinal coherence seems to play a major role in the possible different biological effects of the two sources, as it is associated with the potential for greater penetration of light in tissue/medium.^32^ Different from our data, a previous comparison between laser and LED light did not detect any difference in terms of efficacy,^13^ although this study was performed on MRSA using a different wavelength. The use of blue laser light therefore offers a series of therapeutic opportunities, as it can be efficiently delivered through endoscopic and intravascular catheters and thus reach any internal cavity in the body (e.g., cardiac valves and the urinary tract, which are often contaminated by bacterial biofilms).

While multiple features of laser light, including its monochromaticity, coherence, and unidirectionality, might well explain its superior antimicrobial activity compared to LEDs, the fact that both sources effectively contrast bacteria growth specifically using wavelengths in the blue range point toward the existence of a common molecular target, possibly involving the formation of ROS.

We present different types of indications supporting a role of porphyrins and ROS in the anti-bacterial activity of blue light. Firstly, we measured a significant increase in the level of oxidant species in the medium of bacteria exposed to blue laser irradiation. Secondly, the addition of a potent antioxidant compound, such as ascorbic acid, rescued the viability of blue-irradiated *P. aeruginosa*. Thirdly, a *P. aeruginosa* mutant lacking hemL, a key enzyme in the porphyrin biosynthetic pathway, thus having reduced levels of uroporphyrinogen and protoporphyrin IX,^33–35^ was less sensitive to blue light than wild-type bacteria. While the presence of endogenous porphyrins in *P. aeruginosa* using different techniques has been previously documented using alternative methods,^11,29^ our data provide the first demonstration of their functional involvement as intracellular mediators of the effect of blue light. In line with previous observations,^10^ our data indicate that an important mechanism of the antimicrobial activity of blue light is its capacity to excite porphyrins, which in turn generate ROS, thus killing bacterial cells.

Since our ultimate goal would be the development and optimization of a novel approach to treat skin infections and clear resistant biofilms in vivo, we evaluated the toxicity of several protocols on skin and oral keratinocytes. While TR146 cells did not show any change in cell viability and even increased ATP production shortly after blue light irradiation, HaCaT cells showed a reduction in cell viability only at the highest fluences, supporting the safety of this approach.^11^ Whether the difference between to two cell types was due to either their diverse origin (skin for HaCaT and oral mucosa for TR146 cells) or their different transformation grade (HaCaT are immortalized, whereas TR146 cells are neoplastic) remains an open question.

Based on the previous experiments, we chose protocol A-intermediate (445 nm, irradiance ≤0.30 W/cm^2^, fluence 60 J/cm^2^, CW) for in vivo experiments, ensuring a balance between potent antimicrobial effect and safety on keratinocytes. We confirmed the efficacy of this protocol in decreasing bacterial load at the site of skin infections, both recent or established after 24 h, and limiting the severity of inflammation, consistent with previous studies on *P. aeruginosa* infection treated with blue LED light.^6^ The reduction of inflammation in laser-treated samples also confirms the safety of the approach in terms of possible photothermal damage, as proven by our experiments using a thermographic camera and in accordance with a previous study showing no burn lesions on the skin of healthy volunteers exposed to blue light.^36^

Our results show for the first time that blue laser light effectively inhibits the progression of wound superinfection through the production of intracellular ROS. Additional studies will be needed to prove its potential to completely disrupt established biofilms in vivo, thus setting this approach as a powerful tool to combat *P. aeruginosa* epithelial infection, either alone or as an adjuvant strategy to increase the efficacy of standard antibiotic therapy.

## Methods

### Light sources

A class IV diode laser (K-Laser Blue series, K-laser d.o.o., Sežana, Slovenia) has been used to perform all the experiments, using the protocols described in Table 1. The laser is associated to a programmable scanner conveniently designed to provide uniform irradiation to different multiwell plates, providing blue (445 nm) and infrared (970 nm) wavelength laser light in different combinations of power and energy densities. Plate covers were removed during irradiation and the emission tip was held perpendicular above the cells/animals. The emitted light in continuous wave (CW) completely covered the irradiated field of each culture plate and power was adapted to the spot size to provide the desired irradiance and fluence using an optical power meter (LaserPoint Plus+, Milan, Italy).

Alternative to laser, a LED source providing 380–490 nm light (VALO LED curing light, Ultradent Products, Inc., South Jordan, UT 84095) was employed.

The temperature at the site of irradiation was measured using the thermographic camera Flir One Pro and edited using the software Flir Tools (Flir systems, Antennvägen 6, 187 66 Täby, Sweden).

### Bacteria

*P. aeruginosa* strain ATCC 27853 was purchased from LGC Antimicrobial (LGC, Teddington, United Kingdom) and grown in lysogeny broth (LB broth with agar (Lennox), Sigma Aldrich, Saint Louis, Missouri, U.S.A.) for subsequent laser irradiation. For flow cell experiment, bacteria were cultured in glucose minimal media, obtained by adapting the Jensen’s glucose recipe: NaCl (85.6 mM), K_2_HPO_4_ (14.4 mM), glucose (0.3 mM), MgSO_4_ (1.33 mM), CaCl_2_ (0.14 mM), FeSO_4_ (0.0039 mM), ZnSO_4_ (0.0085 mM), (NH_4_)_2_SO_4_ (15.1 mM).^16^ A *P. aeruginosa* knock-out (KO) mutant (*PA3977*) was purchased from a PAO1 transposon mutant library,^33,35^ available at Manoil Lab (Genome Sciences, University of Washington, Seattle, WA, USA). This mutant lacks the enzyme hemL, which acts in the conversion of glutamate-1-semialdehyde into 2-amino-levulinate during the initial phases of porphyrin biosynthesis,^34^ as indicated in the corresponding KEGG pathway at URL https://www.genome.jp/kegg-bin/show_pathway?pae00860. In order to complement the knock-out mutant (*PA3977*), the complete sequence of the *PA3977* open reading frame together with its ribosome binding site was amplified from *P. aeruginosa* genomic DNA using the Expand High Fidelity PCR System (Roche) and the primers *PA3977* Fw aagcttGCCCGAGAGATAGAGAGCTA (which bears a HindIII restriction site at its 5′) and *PA3977* Rev ggatccGATCATTTCAGCGCGGC (which bears a BamHI restriction site at its 5′). The amplified fragment was purified from an agarose gel using the EuroGOLD Gel Extraction Kit (EuroClone, Milan, Italy) following manufacturer’s instructions, cloned in pGEM-T Easy vector (Promega, Fitchburg, WI, USA) and sequenced at Eurofins Genomics Srl. Having verified the accuracy of the sequence, the *PA3977* fragment was excised from pGEM-T Easy vector using the restriction enzymes HindIII and BamHI (New England Biolabs) and cloned in the corresponding sites of plasmid pBBR MCS-2, in order to get the gene under the control of the Lac promoter. The plasmid obtained (pBBPA3977) was transferred to *PA3977* by triparental mating using *E. coli* (pRK2013) as helper strain and selecting for nitrofurantoin (100 µg/ml) and kanamycin (300 µg/ml).

### Cell lines

Human oral mucosa epithelial cells (TR146, 10032305-1VL, Sigma-Aldrich, Saint Louis, Missouri, U.S.A.) were maintained in Ham’s F12 culture medium, whereas human skin keratinocytes (HaCaT, ATCC PCS-200-011) in DMEM supplemented with 10% (v/v) fetal bovine serum, 100 U/ml Penicillin/Streptomycin, 2 mM Glutamine (Euroclone, Milan, Italy). Cells were seeded at passage 2–8 (10,000 cells/well in 96 multi-well plate and 50,000 cells/well in 24 multi-well-plate) the day before laser irradiation.

### Laser irradiation on planktonic bacteria

Bacteria (10^5^ CFU in 100 μl) were inoculated in 96-well plates, subsequently exposed to multiple protocols (Table 1). The optical density was measured using a spectrophotometer at OD_600_ immediately after irradiation and after 6, 12, 18, and 24 h to evaluate bacterial growth. Protocols A,B,C-high were tested also using infrared laser light. All experiments were performed in biological triplicates.

### Scanning electron microscopy (SEM)

Bacteria were grown for 2 h on sterile 13 mm diameter coverslips (Sarstedt, Newton, NC, U.S.A.) in plates containing 100 μl of LB (10^5^ CFU) and irradiated with protocols A-low, A-intermediate, and A-high, delivering either blue or infrared laser wavelengths. The plates were washed twice with Phosphate Buffer Saline (PBS), fixed with Paraformaldehyde (PFA) 4% (w/v) in PBS for 1 h, dehydrated using ethanol at increasing concentrations (30% (v/v), 50% (v/v), 70% (v/v), 95% (v/v) twice and 100% (v/v) twice −30 min in each solution) and 1,1,1,3,3,3-hexamethyldisilazane (HMDS; Acros Organics, Springfield, NJ, USA) for 90 min. The coverslips were then mounted on metallic stubs, sputter-coated with gold (Sputter Coater K550X, Emitech, Quorum Technologies Ltd, UK) and high resolution SEM images were obtained at different magnifications (×300, ×2400, ×20,000) using a Quanta250 SEM (FEI, Hillsboro, OR, U.S.A.). The working distance and the accelerating voltage were adjusted to obtain the suitable magnification. The ×300 magnification images taken from quadruplicates were analyzed using the ImageJ software (Rasband, W.S., ImageJ, U. S. National Institutes of Health, Bethesda, Maryland, U.S.A., https://imagej.nih.gov/ij/, 1997–2016) to calculate bacteria density. Every image was segmented and the percentage of white pixels, corresponding to the surface covered by bacteria, was calculated.

### Laser irradiation on agar plates

A bacterial suspension (10 μl, 10^6^ CFU/ml) was uniformly swabbed on LB agar Petri dishes and immediately irradiated with protocols A-low and A-intermediate, delivering either blue or infrared laser wavelengths. Bacterial growth was evaluated at 24 h.

### Laser irradiation on microtiter plates

After overnight growth, bacteria culture was adjusted to a 1.0 McFarland nephelometer standard (~corresponding to 3 × 10^8^ CFU/ml) and a 30-fold dilution was obtained. This diluted culture (200 μl) was placed into each well of a 96-well plate, subsequently covered by the peg lid of Calgary biofilm devices (MBEC Assay, Innovotech, Edmonton, Canada). Biofilm was established on the pegs with gentle mixing for 24 h. The device was overturned and each peg was irradiated with blue laser light (protocols A-low, A-intermediate, B-low, and B-intermediate) holding the tip perpendicularly above it. After irradiation, the device was inserted into a recovery plate containing fresh LB growth medium for 24 h. Plates were sonicated at 40 kHz for 10 min to transform biofilms into planktonic cultures and bacterial growth was assessed using an automated plate reader at OD_600_. Values lower than 0.1 were considered as complete eradication of biofilms. Control plates were overturned for the same time of irradiation to mimic the conditions of the treated samples. All experiments were performed in biological triplicates.

### Laser irradiation of biofilms grown in flow cells

Flow cell chambers were inoculated from a mid-log LB culture that was diluted with glucose minimal media to an OD_600_ of 0.05, allowed to attach under static conditions and subsequently cultured in glucose minimal media for 4 days under a constant flow rate (10 ml/h). Both the interference and diffraction of laser light through the plexiglass flow cells were measured using an optical power meter (LaserPoint Plus + , Milan, Italy), revealing and a decrease of ~50% in power density. Therefore, for these experiments, we have indicated the irradiance both at the top and the bottom of the flow cell (protocols A-high and B-high). Laser irradiation, delivering either blue or infrared light, was applied to the central part of each flow cell, containing the mature biofilm. After treatment, biofilms were stained using Syto9 green (5 μM; Life Technologies) to stain the biomass and Propidium Iodide (PI; 30 μM; Life Technologies) to detect extracellular DNA (dead cells) and then visualized using a Zeiss LSM 510 scanning confocal laser microscope (Carl Zeiss Microscopy, Jena, Germany). All experiments were performed in triplicate, acquiring 3 volumetric acquisitions in 3 different areas per sample. Voxel counting was performed using the Volocity software (Improvision, Coventry, England) where Syto9 and PI full Z-stack was measured to encompass the entire biofilms.

### Evaluation of oxidative stress

The Total Oxidant Status (TOS) assay was performed 1 h after irradiation with protocol A-intermediate,^37^ by adding 35 μl of bacterial suspension to Reagent 1 (Reagent 1: xylenol orange 150 μM, NaCl 140 mM, and glycerol 1.35 M in 25 mM H_2_SO_4_ solution, pH 1.75). Subsequently, 11 μl of Reagent 2 (ferrous ion 5 mM and o-dianisidine 10 mM in 25 mM H_2_SO_4_ solution) were added and, after 5 min the absorbance of the mixed solution was measured at OD_560_. All experiments were performed in biological triplicates.

### Cell viability assays

The MTT (Trevigen, Gaithersburg, Maryland, U.S.A.) and ATPlite luciferase (PerkinElmer, Waltham, Massachusetts, U.S.A.) assays were performed on cells seeded in multiwell plates according to manufacturer’s instructions. All experiments were performed in biological triplicates.

### In vivo experiments

Animal care and treatment were conducted in conformity with institutional guidelines in compliance with national and international laws and policies (European Economic Community Council Directive 86/609, OJL 358, December 12, 1987) and upon approval by the Institutional Animal Care Use Committee. 8-week-old adult female C57BL/6 mice were anesthetized and shaved on their right flank. Skin abrasions, measuring 13 mm × 13 mm, were created using a sterile scalpel and topically covered by a *P. aeruginosa* bacterial suspension (10^6^ CFU in 20 μl). To evaluate the effect of blue laser, mice were randomly assigned to either a control or a treatment group, which was irradiated with protocol A-intermediate at 30 min or at 24 h after the infection (*n* = 7 for the control group and *n* = 8 for the treatment group). In both cases, mice were sacrificed after additional 24 h to obtain a square skin specimen (10 mm × 10 mm) corresponding to the wounded and infected area. Every specimen was divided in two identical halves. One half was immersed in 1 ml of LB medium to evaluate bacterial load, by seeding 10 μl of the suspension on agar plates and counting the colonies after 24 h. Results are expressed normalized on control samples. The other half was fixed in PFA 4% (w/v) and 5 μm tissue sections were stained with hematoxylin and eosin for histopathological examination, which was conducted by an expert pathologist (R.B.). The severity of inflammation for each skin layer (epidermis, dermis, hypodermis, panniculus carnosus) was quantified using a semi-quantitative score, described in Table 2.

### Statistical analysis

The Prism 6.0 software (GraphPad Software, La Jolla, CA 92037 USA) was employed for statistical analysis. Friedman’s test and two way ANOVA with post-hoc analysis have been employed to evaluate changes over time for each protocol in the planktonic growth curve experiments, and the Mann–Whitney *U*-test was employed to evaluate differences between treated and untreated samples and at each time point. The Kruskal–Wallis test was used to evaluate differences among groups, and Dunn’s multiple comparison test as post hoc to compare each pair of groups. For in vivo experiments, the Mann–Whitney *U*-test was used to evaluate differences between treated and untreated samples. All statistical assessments were two-sided, and a *p*-value < 0.05 was used for the rejection of the null hypothesis.

### Reporting summary

Further information on research design is available in the Nature Research Reporting Summary linked to this article.

## Supplementary information

Supplementary Information

Supplementary Movie 1

Supplementary Movie 2

Supplementary Movie 3

Supplementary Movie 4

Supplementary Movie 5

Reporting Summary Checklist

## Data Availability

All relevant data used to support the findings of this study are included within the article. Additional information and data are available from the authors upon reasonable request.
