# Supplementary material for: Blue laser light inhibits biofilm formation in vitro and in vivo by inducing oxidative stress
Source: NPJ Biofilms Microbiomes. 2019 Oct 9;5:29. doi: 10.1038/s41522-019-0102-9 (PMC6785554; doi:10.1038/s41522-019-0102-9)
Supplement: Supplementary file 1 — Supplementary Information [file 41522_2019_102_MOESM1_ESM.pdf]

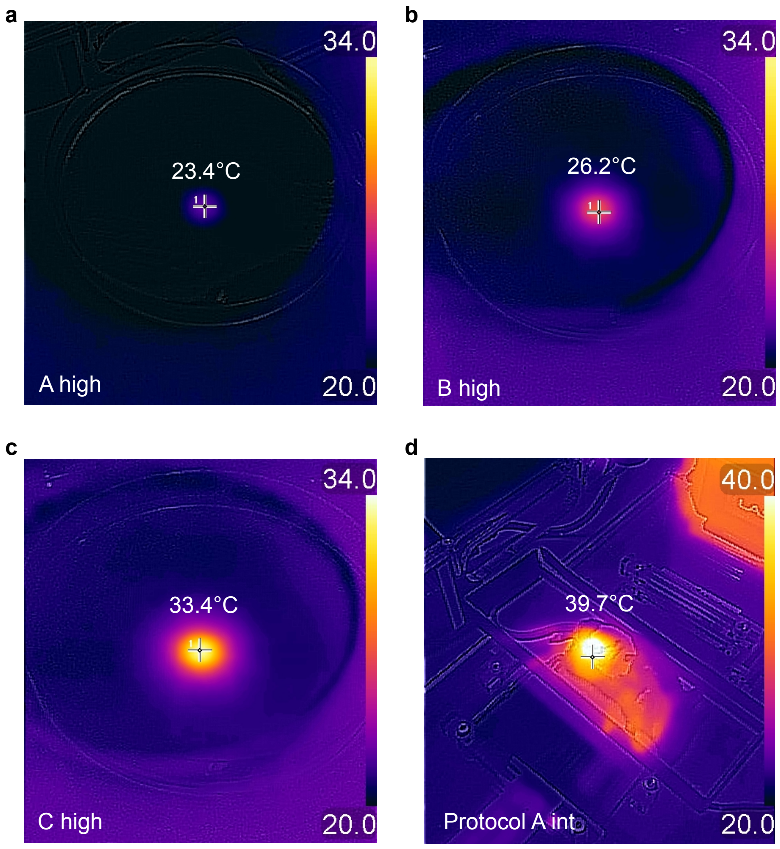


**Supplementary Figure 1**

**Measurement of the temperature at the site of irradiation with different blue laser protocols.**

(a) Temperature measured on a Petri dish with LB agar using protocol A high. (b) Temperature measured on a Petri dish with LB agar using protocol B-high. (c) Temperature measured on a Petri dish with LB agar using protocol C high. (d) Temperature measured on a mouse model of *P. aeruginosa* cutaneous infection, using protocol A-intermediate, used to assess the efficacy of blue laser therapy *in vivo*.

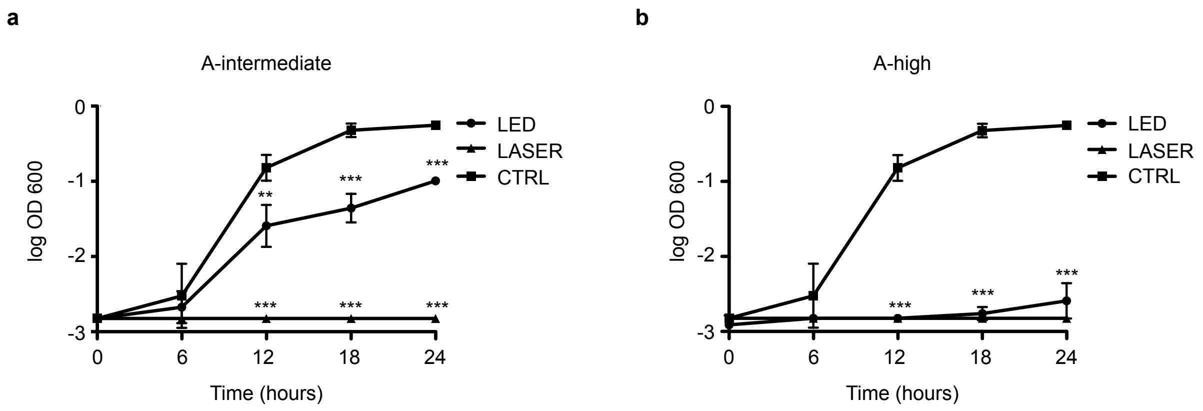


**Supplementary Figure 2**

**Comparison of bacterial growth using either laser or LED light sources.** (a) Planktonic growth curve upon irradiation of *P. aeruginosa* using protocol A-intermediate showing complete inhibition by laser (LT) but not LED sources. ** ANOVA two-way $p < 0.001$; *** ANOVA two-way $p < 0.0001$. (b) Quantification of bacterial growth using protocol A-high (fluence 120 J/cm$^2$) showing complete inhibition by both laser and LED sources. *** ANOVA two-way $p < 0.0001$.

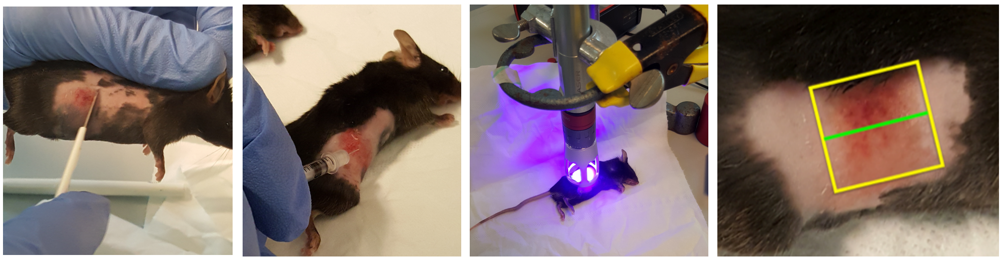

**Supplementary Figure 3**

Representation of the experimental procedure showing skin abrasion, infection with *P. aeruginosa* suspension, exposure to blue laser light (protocol A-intermediate) and tissue harvesting for bacterial load and histological evaluation.
